# Association Between Group Identification at School and Positive Youth Development: Moderating Role of Rural and Urban Contexts

**DOI:** 10.3389/fpsyg.2020.01971

**Published:** 2020-08-07

**Authors:** Diana Paricio, Marina Herrera, María F. Rodrigo, Paz Viguer

**Affiliations:** ^1^Open University of Catalonia, Barcelona, Spain; ^2^Department of Social Psychology, University of Valencia, Valencia, Spain; ^3^Department of Methodology for the Behavioural Sciences, University of Valencia, Valencia, Spain; ^4^Department of Developmental and Educational Psychology, University of Valencia, Valencia, Spain

**Keywords:** adolescence, group identification, peer relationships, positive youth development, rural area, educational intervention

## Abstract

These studies are framed within Social Identity Theory and the Positive Youth Development approach. The aim is: (1) to analyze the relationship between group identification at school and key positive development variables (such as self-esteem, self-efficacy, assertiveness, empathy, alexithymia, satisfaction with life, and academic performance); and (2) examine the moderator role of context (rural or urban areas of residence) and sex in these relationships. The samples were composed of 246 adolescents from a rural context (Study 1) and 156 students from rural and urban contexts (Study 2). As proposed in our hypotheses, the results show statistically significant relationships between group identification and all the variables considered, higher group identification with the class in the rural context, and a moderator role of the context in the relationships between group identification and satisfaction with life, assertiveness, and empathy. These results are relevant for designing and implementing psychoeducational programs to promote positive youth development in both rural and urban contexts.

## Introduction

Despite the importance of the development of social identity during adolescence (Erikson, 1968), a time when interactions with peer groups play an important role in identity formation and adolescent development (Eccles et al., 2003), little attention has been paid to the relationship between social identity and positive youth development (PYD). Although a central aspect of the PYD approach is that it occurs through the interaction between individuals and their social context (Lerner, 2002), most of the research has focused on interpersonal factors, with little attention paid to analyzing the role that groups in general, and social identity in particular, can play in PYD (Bruner et al., 2017). This paper aims to fill this research gap, analyzing the contribution of social identity to PYD.

This contribution may be important if we consider that relevant PYD models include constructs that highlight the role of the individual-social context interaction. For example, the Five Cs model (Lerner et al., 2005) includes the connection competence, which refers to the importance of positive links with other people, including the peer group. In addition, in the model proposed by Oliva et al. (2010), one of the competencies in the area of personal development is the sense of belonging, that is, the degree to which adolescents experience a sense of belonging and satisfaction with their school, as well as the value of the perceived support from teachers as a fundamental part of the link with the school’s teachers.

Social identity is a mechanism through which adolescents establish links and connections with their peers and help them to develop a sense of belonging that can be beneficial to their personal development (Bruner et al., 2017). Psychosocial research has related social identity to a number of variables that are relevant in adolescent development (Bennett and Sani, 2004; Sani, 2012). For example, Haslam et al. (2009) proposed a relationship between group identification, personal development, and positive psychological variables. They emphasize that a sense of shared identity is an important mechanism in making members of disadvantaged groups feel connected, work together, and cope with the negative consequences of their personal circumstances. Based on these assumptions, and in order to advance in this direction, the main purpose of this paper is to examine the association between group identification and central PYD variables.

### Social Identity

According to social identity theory (Tajfel and Turner, 1986; Turner et al., 1987), people can be defined as unique and different from other people (our personal identity – the “self”) or as similar to other people, in terms of being members of groups (our social identity – “us”). Therefore, we can define ourselves as members of a certain in group (e.g., family, school, class). The emotional significance and value that belonging to this group has for the individual is what defines his/her group identification (GI) (Tajfel and Turner, 1986). GI is the feeling of belonging and attachment to the group, along with a feeling of union with the other group members. It is important to highlight that group membership and GI are not synonymous because it is possible to be a member of a group and not feel identified with it (Sani et al., 2012). GI produces a feeling of shared social identity that can have highly significant effects. When we identify with a group, we internalize the norms, values, and beliefs of this group, which affects our attitudes, emotions, and behaviors, and increases the probability of behaving in accordance with the group’s norms (Tajfel and Turner, 1986). Thus, depending on the group’s norms, the consequences of the behavior can be positive (for example, when the functioning norms of this group are favorable to learning and achievement in the academic context), but they can also be negative (for example, when the functioning norms of this group are associated with antisocial or risky behaviors, such as consuming drugs or alcohol). In this regard, a particularly relevant study showed that the relationship between the school climate and students’ behavior problems was mediated by the students’ connection with the school (Loukas et al., 2006). Studies have also found that the students’ perceptions of the school climate contribute to explaining their academic performance, but this effect is mediated by their psychological identification with the school (Maxwell et al., 2017).

This psychological process becomes especially relevant during the adolescent stage because adolescents are more susceptible to being influenced by their peers, and they usually have a strong desire to belong. Some studies show that social identity varies throughout adolescence, and greater identification with the group is found in early adolescence, when teenagers are more concerned with achieving a sense of belonging (Bornholt, 2000; Tanti et al., 2011). They start to understand how important belonging to certain groups is to them (Tarrant, 2002; Bennett and Sani, 2004; Harter, 2012), and they make an effort to understand which groups they identify with (Crosnoe, 2011). Moreover, the majority of adolescents not only identify with groups that share their sociodemographic characteristics (such as age and ethnicity), but also groups that share their activities (for example, from their extracurricular activities or sport clubs) (French et al., 2006; Tanti et al., 2011). Identification with groups of peers has been shown to be important for the psychosocial adjustment of adolescents (Brown and Larson, 2009). Among the groups of peers to which adolescents belong, two are especially relevant: the group of classmates and the group of friends they have outside the school and with whom they spend their free time. Research has shown that identification with these two groups is closely related and contributes greatly to the development of adolescents’ personal identity and their psychosocial adjustment (Alberolo et al., 2018). This relationship is even more significant in the case of adolescents who live in rural areas, to the extent that the group of classmates and the group of friends usually coincide. Moreover, adolescents in rural areas, compared to those in urban areas, have closer social connections and tend to show stronger feelings of bonding and a greater sense of identity, especially with the family and the community (Crockett et al., 2000; Agger et al., 2018).

Therefore, the contribution of social identity to PYD can be especially important in the school environment because it is a favorable context for the development of close social ties and identification with peers, especially in rural areas where the school is the main sphere of interaction for adolescents. Although the relationship between social identity and PYD in the school environment has hardly been analyzed, recent research (Mavor et al., 2017) has shown the importance of the social identity approach in educational practice. Several studies have revealed that the school constitutes a significant psychological group that contributes to the formation of the social identity of its members (Haslam, 2017; Platow et al., 2017). For example, the importance of feeling psychologically connected to the school has been shown in the case of academic achievement (Reynolds et al., 2017a, b). The association between school identification and the development of healthy behaviors in adolescents has also been demonstrated (Miller et al., 2016).

### Positive Youth Development

The present study is also framed within the PYD approach, which emphasizes the adolescent’s emotional, social, and psychological wellbeing. Adolescents are thought to have strengths that can be nurtured, rather than being sources of problems to resolve (Lerner et al., 2005; Benson et al., 2006). The research indicates that interventions based on universal social and emotional learning (SEL) are the best predictors of long-term wellbeing (Taylor et al., 2017). Interventions with adolescents require an understanding of the factors associated with adolescent risk and the ways young people acquire and master the necessary skills to promote healthy development (Brooks-Gunn and Roth, 2014; Ciocanel et al., 2017; Waid and Uhrich, 2020).

There are different conceptual models of PYD. Lerner et al. (2003), in the Five Cs model, identify five groups of elements of this development: *competence, confidence, character, connection, and caring*. In turn, Scales et al. (2000, 2011) propose seven basic elements of positive development: *school success, leadership, valuing diversity, physical health, helping others, delaying gratification, and overcoming adversity*. Oliva et al. (2010) designed a model that defines healthy and PYD, based on 27 specific competencies grouped in five large areas: *personal, social, cognitive, emotional, and moral development.* The competencies related to personal development are located in the center of the model. These are basic competencies, skills, and capabilities that serve as the pillar for the rest of the competencies and, in turn, draw on them. It should be pointed out that there are many similarities between the models, and that important PYD variables, such as self-esteem, self-efficacy, assertiveness, and empathy, are found in both the Five Cs model and in the model proposed by Oliva et al. (2010).

The purpose of the present study is to analyze the relationship between GI and various PYD variables, considering GI as identification with the class group. The PYD variables included in Study 1 stem from both the Five Cs model and the model by Oliva et al. (2010): self-esteem, self-efficacy, assertiveness, and empathy. Moreover, academic performance was added from the Scales et al. (2000) proposal, and satisfaction with life and alexithymia were included because of their relevance in good adjustment in adolescence.

Both self-esteem and self-efficacy are variables included in the Five Cs model, specifically in *confidence*, that is, a positive view of oneself, a sense of self-efficacy, and free will (Lerner et al., 2005), and they are also included in model by Oliva et al. (2010) as part of the personal competences. Self-esteem can be considered one of the most powerful predictors of the degree of psychological adjustment during adolescence (Parra et al., 2004). Self-efficacy has to do with the individual’s perception of his/her ability to achieve an objective. It is also related to life satisfaction, which contributes to the achievement of personal goals, with the resulting benefits (Brannon, 2001).

The assertiveness variable, included in the Five Cs as *competence* (Lerner et al., 2005), is defined as the ability to express oneself and behave efficaciously and appropriately in interpersonal relationships and in diverse contexts. It is also a social competence included in model by Oliva et al. (2010). This variable is important due to the relevant role played by peer interaction in personal and social development during adolescence, a period of profound changes, marked by instability, where relational models are created that later become part of the adult’s personal identity.

Regarding the empathy variable, we employ the definition used by Sánchez-Queija et al. (2006), who consider it a vicarious experience of the emotional state of the other, based on the classic definition by Mehrabian and Epstein (1972). Empathy is defined as a capacity of the individual, almost a personality trait, that has been called dispositional empathy. According to this conceptualization, people will be more or less empathic, without taking into account situational aspects involving physical or relational contexts that generate more or less empathy.

Satisfaction with life is considered the cognitive component of psychological wellbeing and reflects subjective personal wellbeing or the degree to which the individual favorably values his/her quality of life (Reina et al., 2010). Its importance lies in the relationship between satisfaction with life in infancy and adolescence and various indicators of adaptive functioning (Huebner, 2004).

Alexithymia is part of emotional competence, and it is understood as difficulty in recognizing and dealing with our own emotions and identifying those of others (Paricio et al., 2016). Specifically, it is a clinically derived concept that refers to a cognitive–affective disturbance characterized by an individual’s impaired ability to experience, label, and express emotions. Some authors have suggested that the influence of alexithymia on the expression of stress-related pathological states might involve poor resistance to stress. Taking into account that adolescent development is a period of increased susceptibility to stress, low levels of alexithymia may be associated with aspects of functioning associated with self-regulation, mood, and social-emotional development.

Finally, the relationship between GI and academic performance is also analyzed because the latter is one of the main elements of PYD (Scales et al., 2011). Academic performance is integrated into the Five Cs model as *scholastic competence*, which refers to the mastery of certain intellectual and social skills related to good academic performance (Lerner et al., 2005).

As far as we know, only the study by Tarrant et al. (2006) has analyzed the relationship between GI at school and one of the variables involved in PYD, specifically self-esteem. This study highlights the role of identification with the school-based friendship group in self-esteem and various developmental tasks (for example: achieving economic independence). The results show that participants who identified strongly with the group of friends reported higher levels of self-esteem and had a more positive subjective view of personal and social relationships. Other studies referring exclusively to ethnic identity in adolescents have found a positive relationship between this type of GI and self-esteem (Umaña-Taylor, 2004) and between the latter and satisfaction with life (Kiang et al., 2008; Dimitrova et al., 2015). In the school context, some studies show that the feeling of belonging to the school favors students’ wellbeing and commitment (Bizumic et al., 2009; Miller et al., 2015). Moreover, a relationship has been found between GI and some PYD dimensions, such as self-efficacy (Guan and So, 2016) and satisfaction with life (Wakefield et al., 2018), but in an adult population.

Moreover, although there are no conclusive results, the literature shows some sex differences in relevant PYD variables. Thus, for example, research points to greater wellbeing, self-esteem, and psychological adjustment in boys (Pastor et al., 2003; Puskar et al., 2010; Reina et al., 2010), and greater emotional and moral competence in girls (Lerner et al., 2005). Along these lines, Study 1 tested sex differences in PYD variables and GI and the moderator role of sex in the relationships between GI and PYD.

### Rural-Urban Settings and Youth

The role of the rural-urban context in the relationship between GI and PYD has received little attention, although there is evidence of a relationship between social identification and wellbeing in rural areas (Khan et al., 2014). Urban Chinese male adolescents, compared to those in rural areas, have also been found to have a lower sense of family obligation associated with less family identification and lower academic motivation (Fuligni and Zhang, 2004).

However, rural areas also have problems that are often ignored. Contrary to the belief that they avoid the problems and chaos of urban life, rural areas have problems of depopulation, isolation, and low income, which have strong repercussions. Studies show that adolescents who grow up in rural settings, compared to urban settings, are more vulnerable (Elliott and Larson, 2004; Viner et al., 2012; Smokowski et al., 2013; Jiang et al., 2016) and more likely to engage in risk behaviors such as alcohol, tobacco, or other drug use and risky sexual behaviors (Atav and Spencer, 2002). In addition, adolescents in rural settings face more barriers to accessing health resources related to mental and sexual health, including lack of transportation, lack of information about resources, confidentiality concerns, shame, and social stigma (Elliott and Larson, 2004; Curtis et al., 2011).

At the school level, for example, a high prevalence of bullying experiences has been found in rural schools (Dulmus et al., 2006), as well as more school failure compared to suburban areas and impoverished cities in the United States (Provasnik et al., 2007) and a higher incidence of inappropriate behavior during middle school (Witherspoon and Ennett, 2011). In Spain, early adolescents (11 or 12 years old) living in a rural context experience a significant transition from elementary school, a space with a reduced number of classrooms and a reference teacher, to high school, a space with a large number of classrooms and teachers. This transition produces important changes in social experiences (Weiss and Bearman, 2007) and new academic challenges. At the same time, the transition to high school offers new opportunities for extracurricular activities and the chance to develop friendships with more like-minded peers (Sussman et al., 2007).

However, although there is some evidence that school experiences and a sense of worth and belonging to school contribute to predicting academic achievement and adolescents’ aspirations in rural areas (Irvin et al., 2011), research on the importance of this sense of belonging has focused primarily on young people in urban or suburban settings (Irvin et al., 2011), with a shortage of research in rural contexts. In this study, we intend to fill this gap by analyzing the role of sense of belonging and shared identity in PYD in the rural school context.

### The Present Study

The purpose of this study is to examine the association between GI and PYD variables and whether these relationships differ depending on the sex and the context (area where they live: urban or rural); that is, sex and context were tested as moderator variables in Study 1 and Study 2, respectively. Based on previous research, we hypothesized that: (1) there would be significant associations between GI and PYD variables; (2) GI would be higher in adolescents in a rural area; and (3) the associations hypothesized in (1) would be stronger in a rural context. We conducted two studies to test these hypotheses. In Study 1, we examined Hypothesis 1 in a sample of rural students. Moreover, we tested whether sex moderated the relationships between GI and the PYD variables. Study 2 was an extended replication of Study 1, but it included a new sample of rural students, as well as a sample of urban students, in order to determine whether the context plays a moderator role in the relationships between GI and PYD variables.

## Method: Study 1

### Participants

The sample is composed of 246 students in 8th (*N* = 114) and 9th grades (*N* = 130), aged between 12 and 16 years old (*M* = 13.90; *SD* = 0.860); 4.1% of the participants are 12 years old, 27.2% are 13 years old, 46.7% are 14 years old, 19.5% are 15 years old, and 2.4% are 16 years old. Of the total sample, 48.8% are girls, and 51.2% are boys.

The participants belong to three public High Schools (School 1 = 85; School 2 = 60; School 3 = 101) in the province of Teruel, an area in northeastern Spain. All the 8th and 9th grade students in these three schools participated in the study. These high schools were selected through non-probability convenience sampling, and they share the following characteristics: the students who attend these three schools come from rural towns with populations of less than eight inhabitants per km.^2^; they come from towns that are isolated from each other, with populations mainly over 65 years old and a high level of depopulation.

### Measures

#### Group Identification

Tarrant (2002) Group Identification Scale, adapted in Spain by Cava et al. (2011), was used to measure this variable. The scale has 13 items (for example, “I am happy to belong to this class”), with a Likert-type scale (0 = *strongly disagree*, 10 = *strongly agree*). The students received the instruction to respond to the questionnaire by considering the group as the class. The scale had high internal consistency: Cronbach’s α = 0.81.

#### Self-Esteem

To assess self-esteem, the Spanish adaptation by Echeburúa (1995) of the Rosenberg self-esteem scale (RSE; Rosenberg, 1965) was used. It is composed of 10 items (for example, “I think I have a lot of reasons to feel proud”) rated on a Likert-type scale with response options from 1 (*strongly disagree*) to 4 (*strongly agree*). Cronbach’s alpha for this scale in this study was 0.70.

#### Self-Efficacy

The General Self-Efficacy Scale (Baessler and Schwarcer, 1996), validated in Spain by Sanjuán et al. (2000), was used to evaluate this variable. It is a unidimensional scale composed of 10 Likert-type items (for example, “Thanks to my qualities and resources, I can overcome unexpected situations”), where 1 is *strongly disagree* and 4 is *strongly agree*. The internal consistency was α = 0.81.

#### Assertiveness

This was evaluated through Factor 2 of the Social Skills Scale by Oliva et al. (2011) in the Spanish population. It has a Likert-type scale, where 1 is *completely false* and 7 *completely true*, and it is composed of 3 items (for example, “I usually praise or congratulate my classmates when they do something well”). The scale had good internal consistency (α = 0.75).

#### Empathy

To measure this variable, the Basic Empathy Scale by Jolliffe and Farrington (2006), adapted in Spain by Oliva et al. (2011), was used. The adapted scale has 9 items (for example, “Other people’s feelings affect my happiness”), rated on a Likert-type scale (1 = strongly disagree, 5 = strongly agree). Cronbach’s alpha for this scale in this study was 0.75.

#### Satisfaction With Life

To assess this variable, the Satisfaction with Life Scale (SWLS) by Diener et al. (1985), Spanish adaptation by Atienza et al. (2000), was used. It is a unidimensional scale composed of 5 items, where 1 corresponds to “strongly disagree,” and 5 corresponds to “strongly agree” (for example, “If I could live my life over again, I would hardly change anything”). Cronbach’s alpha was 0.80.

#### Alexithymia

To assess this variable, the Toronto Alexithymia Scale (TAS-20) by Bagby et al. (1994), Spanish adaptation by Sánchez-Sosa (2009), was used. It has 20 items (for example, “It is difficult for me to find the right words to express my feelings”), rated on a Likert-type response scale (1 = *strongly disagree*; 6 = *strongly agree*). The scale had high internal consistency: Cronbach’s α = 0.82.

#### Academic Performance

To evaluate this variable, the student’s grade point average from the previous school year, on a scale from 0 to 10, was used. These grades were grouped in 5 response categories: 1 (from 0 to 4.9), 2 (from 5 to 5.9), 3 (from 6 to 6.9), 4 (from 7 to 8.9), and 5 (from 9 to 10).

### Procedure

Participant selection was carried out by means of convenience sampling. The three schools were contacted through a letter of presentation that explained the project and requested a meeting with the school administration. Once these meetings had been conducted, another meeting was held in each school with the entire teaching staff to approve the school’s participation in this study. None of the selected schools refused to participate. The families were informed about the proposed study through a newsletter, and their permission was requested for their children’s participation. The instruments were administered in the students’ usual classroom by a professional who was not associated with the school, for a period of 45 min. The students were informed that their participation in the study was voluntary, anonymous, and confidential. None of the students refused to participate.

### Statistical Analyses

Univariate descriptive analyses were performed, as well as correlation analyses among all the PYD variables considered (self-efficacy, self-esteem, assertiveness, empathy, alexithymia, and satisfaction with life), and between these variables and GI. In addition, the students’ scores on these variables were analyzed to find out whether there were differences depending on sex.

Afterward, to evaluate main and interactive effects of GI and sex, two hierarchical regression models were fitted, for each PYD variable separately. The additive regression model that includes the effects of the sex (0 = female, 1 = male) and GI (continuous variable) variables was fitted in Step 1. To test the moderator role of sex, the multiplicative regression model was fitted in Step 2. In this model, the product of the sex × GI scores (GI × Sex two-way interaction effect) was added to the additive model. This multiplicative model is also called “Moderated multiple regression” (Hayes, 2018), and the focus is on the product term, so that the statistical significance of this term would indicate that sex moderates the relationship between GI and the dependent variable. Although the main focus of the analysis is the significance of the product term in the multiplicative model, the additive model was also estimated because, if this product term is not statistically significant, the unconditional effect of GI should be estimated and interpreted in the additive model (see Hayes, 2018). To test for multicollinearity, the measures of tolerance were obtained. The tolerance values in the additive models were all between 0.98 and 0.99, and so multicollinearity is not a problem in these data. In multiplicative models, the product term is often highly correlated with the independent variables, but this kind of multicollinearity is not considered a problem in moderation tests. Thus, is not necessary, according to the literature, to center the predictors at their means before creating the products to reduce this supposed multicollinearity problem. Moreover, the coefficient regression and *p*-value for the interaction effect remain unchanged whether the predictors are centered or not (Aguinis et al., 2017; Hayes and Rockwood, 2017; Hayes, 2018). All the statistical analyses were performed with the SPSS (v. 24) program.

## Results: Study 1

Descriptive statistics, Pearson’s correlations, and mean differences by sex are presented in Table 1. Regarding the relationships among the PYD variables, statistically significant correlations were observed between the majority of the variables, and they were especially high between self-esteem and self-efficacy (*r* = 0.543; *p* < 0.001) and between self-esteem and satisfaction with life (*r* = 0.464; *p* < 0.001). In the case of academic performance, it was positively and significantly correlated with self-esteem (*r* = 0.154; *p* = 0.016) and with satisfaction with life (*r* = 0.147; *p* = 0.022). With regard to the relationship between GI and the rest of the variables considered, statistically significant positive correlations were found with self-esteem (*r* = 0.169; *p* = 0.008), self-efficacy (*r* = 0.128; *p* = 0.05), assertiveness (*r* = 0.196; *p* = 0.002), empathy (*r* = 0.144; *p* < 0.024), and academic performance (*r* = 0.162; *p* = 0.011). A negative correlation was found with alexithymia (*r* = −0.221; *p* = 0.001), which indicates that greater identification with the class group is associated with less difficulty in identifying and managing one’s own emotions and recognizing those of others.

**TABLE 1:** Bivariate associations among key study variables and covariates in Study 1.

	1		2		3		4		5		6		7		8	
								
	*r* or *M* (*SD*)	*p*	*r* or *M* (*SD*)	*p*	*r* or *M* (*SD*)	*p*	*r* or *M* (*SD*)	*p*	*r* or *M* (*SD*)	*p*	*r* or *M* (*SD*)	*p*	*r* or *M* (*SD*)	*p*	*r* or *M* (*SD*)	*p*
(1) GI	–	–														
(2) Self-esteem	0.169	0.008	–	–												
(3) Self-efficacy	0.128	0.045	0.543	<0.001	–	–										
(4) Assertiveness	0.196	0.002	−0.007	0.912	0.118	0.064	–	–								
(5) Empathy	0.144	0.024	−0.137	0.032	0.084	0.190	0.384	<0.001	–	–	–					
(6) Satisfaction with life	0.111	0.084	0.464	<0.001	0.263	<0.001	0.016	0.806	−0.043	0.499		–				
(7) Alexithymia	−0.221	0.001	−0.235	<0.001	−0.117	0.070	0.051	0.428	0.024	0.709	−0.151	0.018	–	–		
(8) Academic performance	0.162	0.011	0.154	0.016	0.041	0.524	0.111	0.082	0.129	0.044	0.147	0.022	–0.044	0.494	–	–
(9) Sex																
Male	7.49 (1.44)	0.102	3.12 (0.41)	<0.001	3.11 (0.47)	0.002	5.04 (1.18)	0.072	3.51 (0.62)	<0.001	3.79 (0.82)	0.006	3.34 (0.73)	0.763	2.96 (1.02)	<0.001
Female	7.71 (1.27)		2.89 (0.45)		2.93 (0.43)		5.28 (0.87)		3.95 (0.52)		3.48 (0.91)		3.36 (0.68)		3.53 (0.93)	
*M*	7.56		3.01		3.02		5.16		3.73		3.65		3.35		3.24	
*SD*	1.37		0.44		0.46		1.05		0.63		0.88		0.70		1.02	

The *t-*test showed statistically significant differences between the means of boys and girls on self-esteem (*p* < 0.001), self-efficacy (*p* = 0.002), empathy (*p* < 0.001), satisfaction with life (*p* = 0.006), and academic performance (*p* ≤0.001). The means were significantly higher for the boys than for the girls on self-esteem (*M* = 3.12 vs. *M* = 2.89, respectively), self-efficacy (*M* = 3.11 vs. *M* = 2.93, respectively), and satisfaction with life (*M* = 3.79 vs. *M* = 3.48, respectively). However, the boys obtained significantly lower means than the girls on empathy (*M* = 3.51 vs. *M* = 3.95) and academic performance (*M* = 2.96 vs. *M* = 3.53). No statistically significant differences based on sex were observed for GI, assertiveness, or alexithymia.

Table 2 presents the regression analysis for each dependent variable, the *Fs*, *p-*values, and *R*^2^ for the additive model (step 1) and for the multiplicative model, including the interaction effect (Step 2), as well as the regression coefficients of the predictors in the additive model and the product term in the multiplicative model. The GI × Sex interaction effect was not statistically significant in any case (*p’*s > 0.05) (see Step 2 in Table 2), and so sex was not a moderator variable in the relationship between GI and any of the PYD variables. Consequently, the effect of GI must be interpreted from the coefficients in the additive model (Step 1). GI has a statistically significant effect, above and beyond the role of sex, on all the PYD variables (*p’*s < 0.05), and a marginally significant effect on Empathy (*p* = 0.073). These effects were, as expected, all positive, except for alexithymia, which was negative. In other words, higher GI values are associated with higher values on all the PYD variables, and lower values on alexithymia. These models in Step 1 account for significant variance, and the proportion of explained variance (*R*^2^) ranged from 0.047 to 0.133 for satisfaction with life and empathy, respectively.

**TABLE 2:** Additive (step 1) and multiplicative (step 2) regression models for each PYD variable (Study 1).

	*b (SE)*	*p*	*F (df)*	*p*	*R*^2^
Self-esteem					
Step 1			14.381 (2, 242)	<0.001	0.106
Sex	0.25 (0.05)	<0.001			
GI	0.07 (0.02)	0.001			
Step 2					
GI × Sex	−0.01 (0.04)	0.850	9.561 (3, 241)	<0.001	0.106
Self-efficacy					
Step 1			8.018 (2, 242)	<0.001	0.062
Sex	0.20 (0.06)	0.001			
GI	0.05 (0.02)	0.017			
Step 2					
GI × Sex	0.01 (0.04)	0.907	5.328 (3, 241)	0.001	0.062
Assertiveness					
Step 1			6.018 (2, 242)	0.003	0.047
Sex	−0.20 (0.13)	0.130			
GI	0.14 (0.05)	0.004			
Step 2					
GI × Sex	0.08 (0.10)	0.397	4.247 (3, 241)	0.006	0.050
Empathy					
Step 1					
Sex	−0.43 (0.08)	<0.001	18,641 (2, 242)	<0.001	0.133
GI	0.05 (0.03)	0.073			
Step 2					
GI × Sex	0.03 (0.06)	0.573	12.498 (3, 241)	<0.001	0.135
Satisfaction with life					
Step 1			5.964 (2, 241)	0.003	0.047
Sex	0.33 (0.11)	0.003			
GI	0.08 (0.04)	0.041			
Step 2					
GI × Sex	0.08 (0.08)	0.330	4.293 (3, 240)	0.006	0.051
Alexithymia					
Step 1			6.466 (2, 240)	0.002	0.051
Sex	−0.06 (0.09)	0.470			
GI	−0.12 (0.03)	<0.001			
Step 2					
GI × Sex	−0.12 (0.07)	0.068	5.473 (3, 239)	0.001	0.064
Academic performance					
Step 1			12.675 (2, 242)	<0.001	0.095
Sex	−0.54 (0.13)	<0.001			
GI	0.10 (0.05)	0.029			
Step 2					
GI × Sex	0.14 (0.09)	0.134	9.248 (3, 241)	<0.001	0.103

## Method: Study 2

### Participants, Instruments, and Procedure

The sample is composed of 156 students in 8th (*N* = 87) and 9th grades (*N* = 69), with ages between 12 and 16 years old (*M* = 13.72; *SD* = 0.99); 11.5% of the participants are 12 years old, 28.8% are 13 years old, 37.2% are 14 years old, 19.9% are 15 years old, and 1.9% are 16 years old. Of the total sample, 46.8% are girls, and 53.2% are boys. The participants are from two public high schools (ESO), one located in a rural context in the province of Valencia (rural high school = 85), and the other located in an urban context, specifically in Valencia, the third city in Spain in terms of density (urban high school = 71). The two high schools were selected through non-probability convenience sampling. All the 8th and 9th grade students in the rural school participated in this study. In the urban school, the convenience sample was selected by the school’s management team, using the inclusion criterion that students had to be in 8th or 9th grade. The same variables were measured by the same scales as in Study 1, except alexithymia.

### Statistical Analyses

First, sample descriptive statistics and correlations between variables were examined. Bivariate analyses were conducted using *t*-tests for categorical independent variables, and Pearson’s correlations for continuous variables. Bivariate analyses were used to examine zero correlations between the PYD variables and GI to explore mean differences in the study variables according to sex and context. Afterward, to evaluate main and interactive effects of GI and context, controlling for sex, two hierarchical regression models were fitted, as in Study 1, for each PYD variable separately. The additive model that includes the effects of the sex (0 = female, 1 = male), context (0 = urban, 1 = rural), and GI (continuous variable) variables was fitted in Step 1. The multiplicative model, in which the product of the scores for context x GI (GI × Context two-way interaction effect) was added to the additive model, was fitted in Step 2. The focus here is on the product term, so that the statistical significance of this term would indicate that context moderates the relationship between GI and the dependent variable. To test for multicollinearity, the measures of tolerance were obtained. The tolerance values in the additive models were all between 0.84 and 0.99, and so multicollinearity is not a problem in these data. As in Study 1, multicollinearity in the multiplicative models is not considered a problem, and predictors were not centered at their means. Analyses were conducted using SPSS (Version 24.0). In the case of a statistically significant GI × Context interaction, planned *post hoc* simple slope analyses were performed to estimate conditional effects of GI on the PYD variables in rural and urban contexts using the PROCESS macro (Hayes, 2018).

## Results: Study 2

Descriptive statistics and results of the bivariate analysis are presented in Table 3. GI had a statistically significant positive correlation with self-esteem, assertiveness, satisfaction with life, and empathy (*r*’s from 0.246 to 0.459; *p’*s < 0.05), and marginally significant with self-efficacy (*r* = 0.156; *p* = 0.058). Regarding differences by sex, boys reported higher satisfaction with life than girls (*M* = 4.06 vs. *M* = 3.53; *p* < 0.001). For the context variable, GI means were significantly higher in the rural context (*M* = 7.69 vs. *M* = 6.36; *p* < 0.001). Context was also significantly associated with assertiveness and empathy, with higher means on the two variables in the rural context (*p* < 0.05).

**TABLE 3:** Bivariate associations among key study variables in Study 2.

	1		2		3		4		5		6		7	
								
	*r* or *M* (*SD*)	*p*	*r* or *M* (*SD*)	*p*	*r* or *M* (*SD*)	*p*	*r* or *M* (*SD*)	*p*	*r* or *M* (*SD*)	*p*	*r* or *M* (*SD*)	*p*	*r* or *M* (*SD*)	*p*
(1) GI	–	–												
(2) Self-esteem	0.376	<0.000	–	–										
(3) (Self-efficacy	0.156	0.058	0.287	0.001	–	–								
(4) Assertiveness	0.246	0.002	0.182	0.031	0.203	0.013	–	–						
(5) Empathy	0.191	0.019	0.024	0.777	0.092	0.266	0.439	<0.001	–	–				
(6) Satisfaction with life	0.459	<0.000	0.455	<0.001	0.397	<0.001	0.412	<0.001	0.205	0.012	–	–		
(7) Academic performance	−0.013	0.877	0.016	0.855	0.172	0.037	–0.021	0.800	−0.034	0.679	0.036	0.659	–	–
(8) Sex														
Male	7.24 (1.81)	0.296	2.64 (0.31)	0.235	3.04 (0.38)	0.570	5.51 (1.06)	0.281	3.70 (0.57)		4.06 (0.70)		3.06 (1.13)	
Female	6.93 (1.88	<0.000	2.58 (0.28)		3.01 (0.49)		5.33 (1.03)		3.76 (0.57)	0.562	3.53 (0.88)	<0.001	3.33 (1.04)	0.127
(9) Context														
Rural	7.69 (1.78)		2.58 (0.31)	0.084	3.03 (0.46)	0.956	5.62 (1.11)	0.011	3.82 (0.62)		3.87 (0.93)		3.08 (1.04)	0.188
Urban	6.36 (1.66)		2.67 (0.29)		3.02 (0.41)		5.19 (0.92)		3.62 (0.47)	0.035	3.75 (0.69)	0.398	3.32 (1.16)	
*M*	7.10		3.01		3.02		5.43		3.73		3.82		3.19	
*SD*	1.84		0.44		0.44		1.05		0.56		0.83		1.09	

Table 4 presents the regression analysis for each dependent variable, the *Fs*, *p*-values, and *R*^2^ for the additive model (step 1) and for the multiplicative model, including the interaction effect (step 2), as well as the regression coefficients of the predictors in the additive model and the GI × Context interaction in the multiplicative model. The regression coefficients of the interaction were statistically significant for the dependent variables of empathy (*p* = 0.009) and satisfaction with life (*p* = 0.014), and marginally significant for assertiveness (*p* = 0.085). The proportion of explained variance (*R*^2^) for these multiplicative models was 0.094 (for empathy), 0.326 (for satisfaction with life), and 0.102 (for assertiveness).

**TABLE 4:** Additive (step 1) and multiplicative (step 2) regression models for each PYD variable (study 2).

	*b (SE)*	*p*	*F (gl)*	*p*	*R*^2^
Self-esteem					
Step 1			7.591 (3, 138)	<0.001	0.142
Sex	0.01 (0.07)	0.897			
GI	0.09 (0.021)	<0.001			
Context	0.00 (0.08)	0.996			
Step 2					
GI × Context	−0.004 (0.04)	0.923	5.654 (4, 137)	<0.001	0.142
Self-efficacy					
Step 1			1.416 (3, 144)	0.241	0.029
Sex	0.04 (0.07)	0.607			
GI	0.04 (0.02)	0.056			
Context	−0.05 (0.08)	0.546			
Step 2					
GI × Context	−0.03 (0.04)	0.508	1.168 (4, 143)	0.327	0.032
Assertiveness					
Step 1			4.524 (3, 149)	0.005	0.083
Sex	0.15 (0.17)	0.359			
GI	0.11 (0.05)	0.026			
Context	0.30 (0.18)	0.093			
Step 2					
GI × Context	0.17 (0.10)	0.085	4.188 (4, 148)	0.003	0.102
Empathy					
Step 1			2.589 (3, 147)	0.055	0.050
Sex	−0.06 (0.09)	0.518			
GI	0.05 (0.03)	0.073			
Context	0.13 (0.09)	0.194			
Step 2					
GI × Context	0.14 (0.05)	0.009	3.773 (4, 146)	0.006	0.094
Satisfaction with life					
Step 1			20.922 (3, 148)	<0.001	0.298
Sex	0.46 (0.12)	<0.001			
GI	0.22 (0.03)	<0.001			
Context	−0.19 (0.12)	0.130			
Step 2					
GI × Context	0.17 (0.07)	0.014	17.765 (4, 147)	<0.001	0.326
Academic performance					
Step 1					
Sex	−0.26 (0.18)	0.155	1.260 (3, 147)	0.291	0.025
GI	0.02 (0.05)	0.678			
Context	−0.25 (0.19)	0.200			
Step 2					
GI × Context	−0.16 (0.11)	0.124	1.554 (4, 146)	0.190	0.041

**FIGURE 1:**
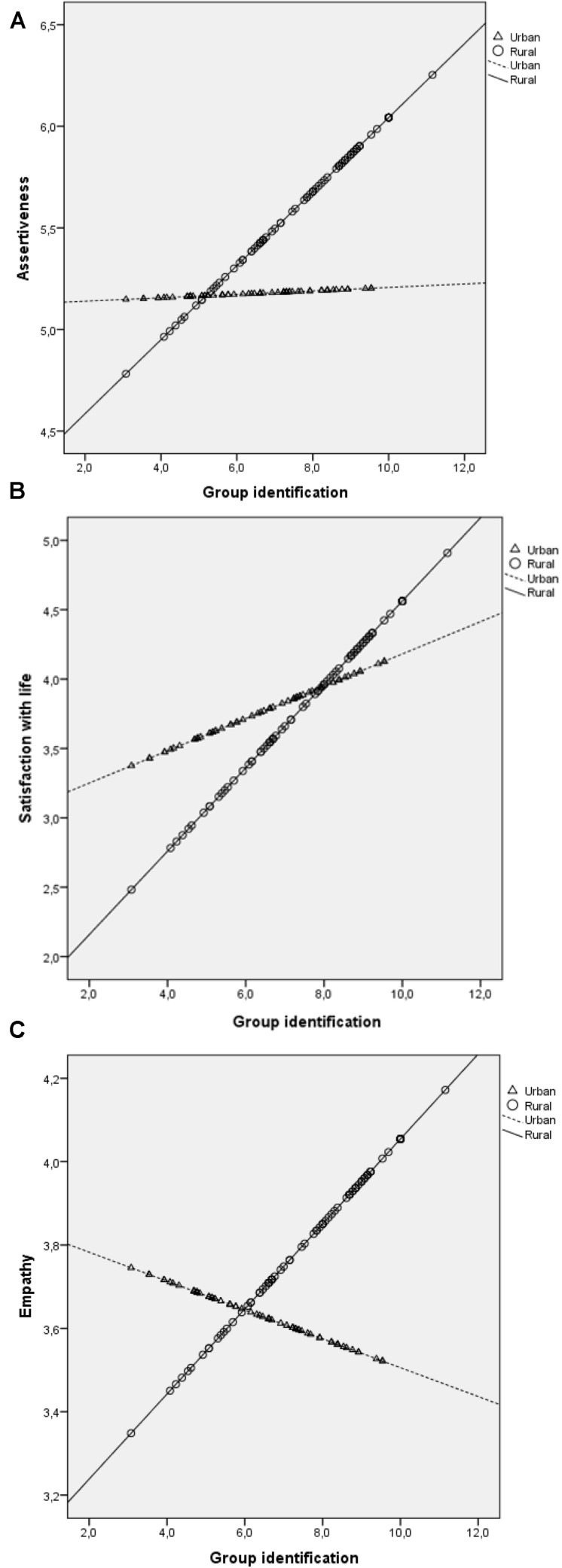
*Post hoc* simple slope analyses: **(a)** Interaction of group identification and context on assertiveness. **(b)** Interaction of group identification and context on satisfaction with life. **(c)** Interaction of group identification and context on empathy.

Planned *post hoc* simple slope analyses were performed on the three dependent variables (Figure 1). Regarding empathy, the analysis of the slopes for each group indicated that the association between GI and empathy was statistically significant only in the rural context (β urban = −0.03, *p* = 0.39; β rural = 0.104, *p* = 0.002). As for satisfaction with life, the slopes in both groups were statistically significant (β urban = 0.119, *p* = 0.02; β rural = 0.285, *p* < 0.001) and in the same direction. Thus, the significant interaction indicates that the association between GI and satisfaction with life is stronger in the rural context. Finally, for assertiveness, the association between GI and assertiveness was statistically significant only for the rural group (β urban = 0.01, *p* = 0.92; β rural = 0.17, *p* = 0.005).

For the other dependent variables, the interaction effect was not statistically significant, and so the effect of GI must be interpreted from the coefficients in the additive model (step 1). In predicting self-esteem, variables entered in step 1 accounted for significant variance (*F*[3,138] = 7.591, *p* < 0.001, *R*^2^ = 0.142), and GI was a statistically significant predictor (*p* < 0.001). Regarding self-efficacy, the variables entered in step 1 did not account for significant variance (*F*[3,144] = 1.416, *p* = 0.241, *R*^2^ = 0.029), but the effect of GI was marginally significant (*p* = 0.056). Finally, for academic performance, the model in step 1 did not account for significant variance (*F*[3,147] = 1.260, *p* = 0.291, *R*^2^ = 0.025).

## Discussion

This paper furthers our understanding of the role of GI at school in PYD, as well as the potential moderating role of the sex and context (rural vs. urban) variables in this association. It not only contributes to showing the importance of groups of peers at school in adolescent development in rural contexts, but it also reveals the importance of the social identity perspective in this development.

In line with our first hypothesis regarding the positive association between GI and PYD, our two studies show a relationship between GI and self-esteem, self-efficacy, assertiveness, empathy (marginally significant in Study 1), satisfaction with life, and academic performance (in Study 1), and a negative relationship with alexithymia (only in Study 1). The results confirm the relationship between GI and self-esteem obtained by Tarrant et al. (2006) and reveal new relationships with other variables of positive adolescent development. To the best of our knowledge, the relationships between GI and these PYD variables have not previously been studied in adolescents, and they show the importance of identification with the school in general, and with the group of classmates in particular, in the development of the main variables proposed by the different models (such as self-efficacy, assertiveness, and empathy) as contributing to the emotional, social, and psychological wellbeing of the adolescent. Furthermore, our results from Study 1 provide new evidence about the gender differences in adolescents, with boys obtaining better results on self-esteem (Quatman and Watson, 2001) and self-efficacy, and girls obtaining better results on empathy (Sánchez-Queija et al., 2006) and academic performance (Hernando et al., 2012). However, sex did not moderate the relationships between GI and the PYD variables; that is, these relationships were found to have the same magnitude in boys and girls.

Regarding the relationship between GI and academic performance, this study shows that students who identify more with their peer group report greater academic performance. Although this relationship is only found in Study 1, the result is relevant because, according to Scales et al. (2011) and the Five Cs model, school success is one of the fundamental elements of positive development in adolescents. Therefore, the role of GI should be considered, as well as the developmental assets pointed out in the literature (Scales et al., 2000), when predicting academic success. However, there is a need for more studies that contribute to clarifying the contribution of this variable to predicting academic performance. As in the case of the PYD variables, sex did not moderate the relationship between GI and academic performance.

Supporting our second hypothesis, findings from our second study showed that GI with the class was higher in the rural context than in the urban context. This result is consistent with the limited evidence available, which shows that adolescents from rural areas tend to show a greater feeling of identity, closer social connections, and greater social responsibility (Crockett et al., 2000; Johnson et al., 2005; Agger et al., 2018). In contrast to the anonymity of urban areas, in rural areas there is a culture based on co-existence, where the levels of solidarity, social support, and integration and the psychological sense of community tend to be greater (Roussi et al., 2006; Berry and Okulicz-Korzaryn, 2009).

Our third hypothesis was partially supported by showing that the effect of the GI × context interaction was significant for empathy and satisfaction with life, and marginally significant for assertiveness. In the case of self-esteem, there was a main effect of GI, but this effect was not moderated by the context. With regard to assertiveness and empathy, the association between GI and these two variables was only significant in the rural group. Undoubtedly, both variables are important in positive adolescent development because they are included in most of the existing models. Our study indicates that these two variables are even more important in the rural context. It seems logical that if a person defines him/herself positively within a group, he/she will also have a greater feeling of affinity at a dispositional level and show more empathy and a more favorable attitude toward the members of the group.

The variance in satisfaction with life explained by the GI × Context interaction was especially high, which suggests that stronger social connections and cooperation networks in rural areas can be one of the reasons for greater satisfaction with life. This is especially relevant if, as shown, satisfaction with life is significantly related to commitment at school (Awang-Hashim et al., 2015). This finding is consistent with previous studies suggesting that people in rural areas are more satisfied with their lives than people in urban areas. For example, using data from the 2008 European Study of Values to analyze the differences in satisfaction with life in urban and rural areas of the European Union, Sorensen (2014) found that satisfaction is greater in the latter. Studies have also shown that adolescents from urban areas experience high levels of loneliness and low levels of satisfaction with life (Okwaraji et al., 2018), and that satisfaction with life is related to a sense of community in rural areas (Prezza and Constantini, 1998). This sense of community may contribute to greater wellbeing, stimulate a stronger feeling of identity, and facilitate social relations in rural areas. Moreover, as our results indicate, there is evidence showing gender differences in adolescents’ satisfaction with life, with boys exhibiting higher levels of life satisfaction (Moknes and Espnes, 2013) and a greater relationship between collective self-esteem and satisfaction with life (Zhang and Leung, 2002).

These studies have several limitations. First, the two studies presented are cross-sectional and do not allow us to draw causal inferences. Future work should evaluate the effect of GI on PYD variables using (quasi) experimental studies or longitudinal methods to provide stronger evidence and clarify these relationships. Second, we found contradictory results for the relationships between academic performance and GI in Studies 1 and 2. Given the relevance of this variable, future research should further examine this relationship by including different measures and multiple dimensions of academic performance. Third, the samples were convenience samples and limited in size (mainly the sample for Study 2), and they came from specific areas in Spain; thus, it remains unclear whether our results could be generalized to other rural and urban areas that differ from those of the current samples. Finally, it would be interesting to test the relationships between GI and the PYD variables (and academic achievement), not only in adolescents, but also in children (Bennett and Sani, 2004).

## Conclusion

Despite these limitations, the present study has several strengths and extends previous literature by showing that groups play an important role in people’s lives, especially in adolescence, when peer groups are fundamental to the formation of identity and optimal youth development. This study provides empirical evidence for the role social identity plays in PYD, and it advances the understanding of this relationship. The results show that identification with the class is important for psychological development in the scenario of change and instability associated with adolescence. Its contribution is especially relevant in the rural context; whose characteristics make the adolescent more vulnerable. Therefore, as this study shows, identification with the class can be a mechanism to help adolescents in rural areas to feel united, achieve a shared identity, and cope with their particular circumstances.

Overall, our results suggest that adolescents’ positive development and academic performance could be fostered by implementing psychoeducational programs that strengthen their GI with their classmates, especially in rural areas. Future studies will be oriented toward fostering youths’ positive development through educational interventions that provide adolescents with opportunities to develop a sense of belonging to the school and the class, as well as positive social norms (see, for example, Haslam et al., 2016; Scarf et al., 2017).

## Data Availability Statement

The raw data supporting the conclusions of this article will be made available by the authors, without undue reservation.

## Ethics Statement

These studies were carried out in accordance with the recommendations of the Code of Ethics of the University of Valencia. Informative meetings were held with the selected schools management teams to explain the objectives and methodology of the studies. The management teams of the five schools approved their participation in the studies and gave their written consent. Subsequently, all the parents gave written informed consent for the adolescents’ participation in the studies. In addition, the students were informed that their participation in the study was voluntary, anonymous, and confidential. None of the students refused to participate. This study followed the ethical values established in the 1964 Declaration of Helsinki and its later amendments and the UNESCO Universal Declaration of Human Rights.

## Author Contributions

DP, MH, MR, and PV designed the study. DP collected the data. DP and MR analyzed the data. All the authors wrote and revised the manuscript. MH, MR, and PV supervised the project.

## Conflict of Interest

The authors declare that the research was conducted in the absence of any commercial or financial relationships that could be construed as a potential conflict of interest.
